# Plasticity of Root Architecture and ROS–Auxin Regulation in *Paeonia ostii* Under Root-Zone Restriction

**DOI:** 10.3390/plants14121889

**Published:** 2025-06-19

**Authors:** Qiang Xing, Ruotong Zhao, Peng Zhou, Jun Qin, Heming Liu, Shuiyan Yu, Bin Zhao, Yonghong Hu

**Affiliations:** 1 School of Life Science, Fudan University, Shanghai 200433, China; xingqiang0731@126.com; 2 Shanghai Chenshan Botanical Garden, Shanghai 201602, China; z1364181613@163.com (R.Z.); qinjun03@126.com (J.Q.); liuheming@csnbgsh.cn (H.L.); yushuiyan1982@163.com (S.Y.); 3 College of Landscape Architecture and Art, Fujian Agricultural and Forestry University, Fuzhou 350002, China; 4 College of Agriculture and Biology, Shanghai Jiao Tong University, Shanghai 200240, China; pzhou0063@sjtu.edu.cn

**Keywords:** root morphology, biomass, antioxidant enzymes, gene expression, auxin signaling, horticultural optimization

## Abstract

Root zone restriction (RZR) technology optimizes plant growth and quality. However, the fleshy root system of *Paeonia ostii* exhibits sensitivity to spatial constraints, and research on the plasticity of its root architecture and adaptation mechanisms remains inadequate. This study provides a functional analysis of biomass allocation and root architectural responses to the root-zone restriction (RZR) in *P. ostii*, comparing three container volumes (8.5, 17, and 34 L). While the total biomass increased with root zone volume (e.g., shoot biomass rose from 9.30 g to 59.94 g), RZR induced a 44.8% increase in root-to-shoot ratio, indicating carbon reallocation to enhance belowground resource acquisition. The principal component analysis identified root biomass, volume, and surface area as key plasticity drivers. Optimal root efficiency occurred at 26.09–28.23 L, where root length and tip/fork numbers peaked. Mechanistically, RZR elevated superoxide dismutase (SOD) activity by 49.74% but reduced catalase (CAT) by 74.24%, disrupting H_2_O_2_ homeostasis. Concurrently, auxin transporter genes (*PIN1*, *AUX1*) were upregulated, promoting root elongation and lateral branching through auxin redistribution. We hypothesize that ROS–auxin crosstalk mediates architectural reconfiguration to mitigate spatial stress, with thickened roots enhancing structural stability in restricted environments. The study underscores the need to optimize root zone volume in woody species cultivation, providing thresholds (e.g., >28 L for mature plants) to balance biomass yield and physiological costs in horticultural management.

## 1. Introduction

The effects of root-zone restriction on biomass allocation and root system architecture in *Paeonia ostii*, represent a critical area of study in plant physiology and ecology. Research in this field is not only related to the improvement of crop yields and the optimization of crop quality, but also has a profound impact on the improvement of human living quality [1]. As the national flower of China, peony has important ornamental value and cultural significance, and occupies an important position in the fields of horticulture, ecological environment restoration, etc. Firstly, peony is popular due to its diverse flower forms and rich flower colors, and is also widely used in the fields of garden landscape design and ecological environment restoration because of its strong adaptability. Secondly, peony seed oil is rich in unsaturated fatty acids (accounting for about 92%), among which the content of α-linolenic acid is as high as 38–42% [2], which is significantly higher than that of traditional oil crops. Its unique compounds, such as fatty acids, paeoniflorin, and paeonol, hold the potential for regulating lipid metabolism and improving cardiovascular function [3]. Its remarkable antioxidant activity also provides a new source for the development of natural antioxidants [4]. As a woody ornamental plant of high economic value, root system development is a critical factor influencing its growth and functional value [5,6,7]. The root system supports the growth and development of the aboveground parts by absorbing water and nutrients.

Root zone restriction is a cultivation technique that physically or ecologically confines plant roots within a defined soil volume to regulate plant growth and development. It plays a significant role in improving resource use efficiency, promoting plant productivity, and enhancing the quality of agricultural products, making it an important theoretical foundation for achieving precision agriculture and sustainable development [8]. As a widely adopted cultivation practice, root zone restriction exerts notable effects on plant growth, physiological processes, and product quality by artificially limiting root spatial expansion. In terms of growth, it suppresses vegetative development while promoting reproductive growth, thereby improving fruit quality and yield [8,9,10]. Physiologically, it influences photosynthetic efficiency and alters hormonal balance, ultimately affecting plant development [11,12]. In terms of quality, it can enhance the soluble sugar content and sweetness of fruits, improve their external appearance, and strengthen stress resistance, enabling plants to better survive and grow under adverse conditions [13,14]. In recent years, root zone restriction has been widely applied in plant growth regulation and production management, particularly in economically important crops such as grape, pear, peach, and tomato [8,11,13,15]. Studies have shown that appropriate root zone restriction contributes to regulating root development, improving nutrient uptake efficiency and water use, thereby increasing the aboveground biomass and economic value. These findings offer valuable references for the cultivation of *Paeonia* spp. Peony possesses a fleshy root system, which differs markedly from woody or fibrous root systems in terms of nutrient absorption and spatial adaptability [16]. Fleshy roots are generally more sensitive to spatial constraints, leading to reduced nutrient uptake capacity and subsequently impaired shoot growth [16]. This characteristic makes the study of peony under root zone restriction particularly relevant. However, existing research has primarily focused on peony’s cultivation techniques [17], stress resistance [18], and ornamental traits [19,20], while studies on its root plasticity and adaptive mechanisms under spatial constraints remain limited. This research reveals the three-dimensional plasticity response of *P. ostii*’s fleshy roots under root confinement. It quantifies the optimal peak range of root architecture and elucidates the novel ROS–auxin interplay mechanism driving these morphological adaptations. Through this study, we aim to propose an innovative stage-specific container modulation strategy, thereby filling critical gaps in the spatial adaptation theory for succulent-rooted plants.

While root-zone restriction has been widely studied and applied in cash crops [21,22], research on *Paeonia* spp.—a vital horticultural plant—remains limited. Specifically, there is a lack of systematic investigation into the relationship between root architecture, root biomass, and root-zone restriction, as well as insufficient quantitative screening for the optimal root-zone volume. The mechanisms by which root-zone restriction influences root architecture and biomass accumulation in *P. ostii*, thereby regulating whole-plant physiological and ecological processes, and the role of the antioxidant–auxin regulatory network in these responses remain uncharacterized, with limited experimental evidence. Therefore, this study investigated the effects of root-zone restriction on root architectural traits and root biomass morphology in *P. ostii* using different container sizes. By integrating measurements of antioxidant enzyme activities and key gene expressions associated with root development, we explored the comprehensive impacts of root-zone space on plant growth. These findings contribute to a deeper understanding of growth response mechanisms in plants under limited spatial conditions, provide theoretical support for elucidating the physiological adaptation mechanisms of *P. ostii* in restricted root zones, and offer foundational data for the development and utilization of *P. ostii* as a biomass energy material.

## 2. Results

### 2.1. Effects of Root-Zone Restriction on Biomass Allocation in P. ostii

**Figure 1:**
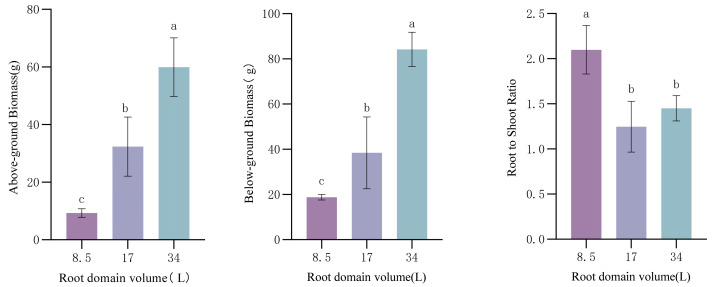
Effect of root-domain limitation on *Paeonia ostii* biomass. Different lowercase letters indicate significant differences in plant biomass in different root domain volume at 0.05 level.

In the three treatments, the patterns of aboveground and underground biomass changes were generally consistent, with the dry matter weights of various plant parts increasing significantly with increasing root-zone volume (*p* < 0.05). At a root-zone volume of 8.5 L, both aboveground and underground biomass were significantly lower than those in the other two groups (Figure 1). The root–shoot ratio (RSR) in the small pot was significantly higher than in the other treatments, with the medium pot showing the smallest value, and significant differences existed among the three treatments (Figure 1). In the small pots, the root system developed numerous root tips and forks to enhance water and nutrient absorption, while aboveground biomass decreased due to root-zone restriction, leading to an increased numerator (root dry weight) and decreased denominator (shoot dry weight) in the RSR ratio. At the end of the full-bloom stage in May, when aboveground biomass reached its maximum, the RSR ratio was minimized. Under small-container conditions, root-zone restriction reduced flowering and leaf expansion, resulting in a significantly higher RSR ratio compared to medium and large pots.

### 2.2. Effects of Root-Zone Restriction on Root Architecture of P. ostii

**Figure 2:**
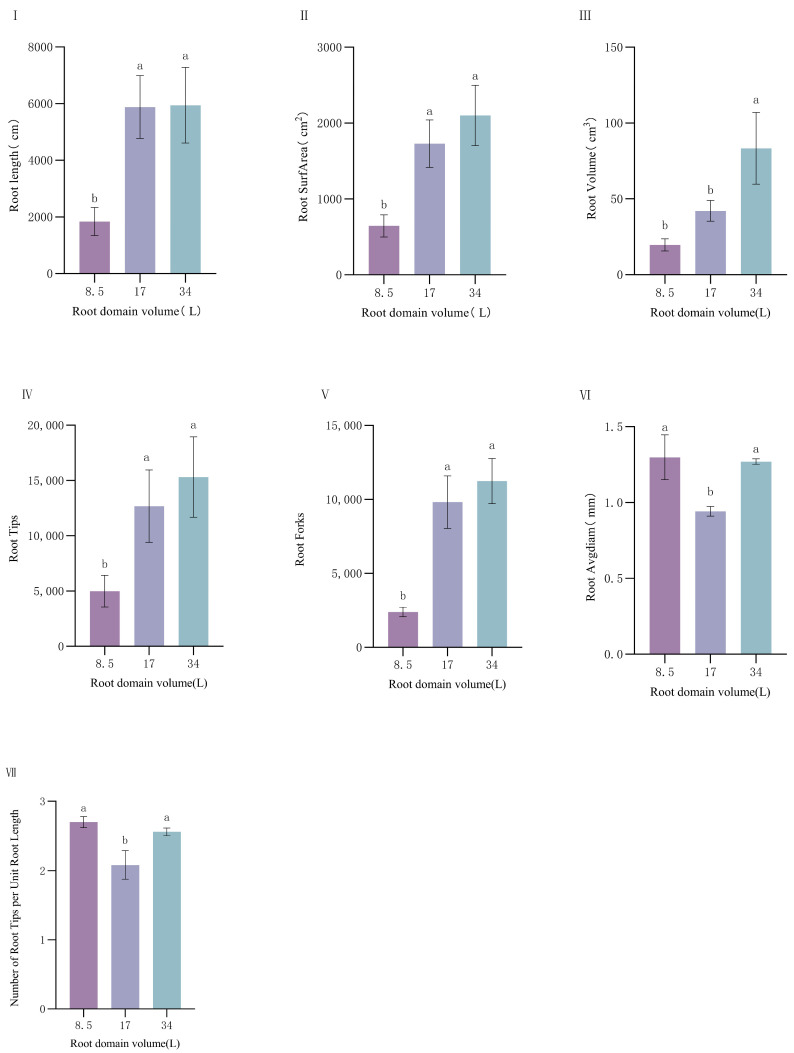
Influence of root-domain restriction on *P. ostii* root conformation. (**I**) root length; (**II**) root surface area; (**III**) root volume; (**IV**) number of root tips; (**V**) number of root forks; (**VI**) average root diameter; (**VII**) number of root tips per unit root length. Different lowercase letters indicate significant differences in plant root conformational characteristics in different root domain volume at 0.05 level.

As shown in Figure 2, root-zone restriction exerted significant effects on root growth. Parameters including root length (RL), root surface area (SA), root volume (RV), number of root tips (RT), and number of root forks (RF) followed a consistent pattern: large pot > medium pot > small pot, indicating that larger root-zone space effectively supports root expansion and development, significantly enhancing the plant’s resource acquisition capacity. Average root diameter (RD) showed a trend of small pot > large pot > medium pot, with no significant difference between the large and small pot treatments (*p* < 0.05). This suggests that under restricted root-zone conditions, *P. ostii* enhances structural support by increasing root diameter to adapt to environmental stress in limited space. There was no significant difference in the number of root tips per unit root length between the plants in small pots and those in large pots. This suggests that under the condition of a restricted root zone, plants compensate for the insufficient root space by promoting the formation of lateral roots and increasing the number of root tips so as to enhance their ability to absorb external resources.

### 2.3. Effects of Root-Zone Restriction on Morphological Traits of P. ostii

#### 2.3.1. Characteristics of Underground Root Architecture and Biomass Variability

Descriptive analysis of biomass and root parameters in *P. ostii* revealed that (Table 1) aboveground biomass (AGB) and root volume (RV) had the highest coefficients of variation (CV), at 74.68% and 73.01%, respectively, followed by underground biomass (RB, 69.7%). The lowest CV was observed for average root diameter (AD, 18.48%). High CV values indicated greater sensitivity of these growth indices to root-zone restriction. The order of variability among parameters was AGB > RV > RB > RF > RT > RL > SA > RSR > AD. The high CVs of AGB and RB suggested substantial differences in aboveground and underground biomass under varying root-zone spaces or conditions, reflecting adaptive adjustments in plant resource allocation. With a CV of only 18.48%, AD was significantly lower than other parameters, indicating that *P. ostii* may maintain a relatively stable root diameter to adapt to restricted root zones, ensuring structural support and absorption efficiency in limited space. In terms of root architecture, parameters such as RL, SA, RV, RT, and RF exhibited high CVs (exceeding 50%), particularly RV and RL. These highly variable indices demonstrated significant changes in root structural traits under different environments, highlighting the plasticity of plant roots under root-zone restriction.

#### 2.3.2. Correlation Between Root-Zone Volume and Characteristics of Underground Root Architecture and Biomass

**Figure 3:**
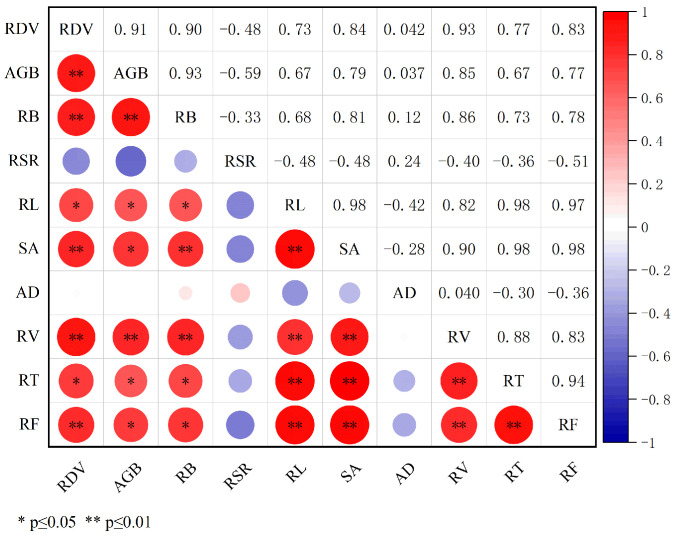
Correlation between conformation characteristics and biomass of underground root systems. The color gradient from blue to red represents the correlation direction and strength: blue for negative correlations and red for positive correlations, with the color intensity and circle size jointly reflecting the correlation magnitude. **. Correlation is significant at the 0.01 level. *. Correlation is significant at the 0.05 level. RZV = root-zone volume; AGB = aboveground biomass; RB = root biomass; RSR = root–shoot ratio; RL = root length; SA = root surface area; AD = average root diameter; RV = root volume; RT = number of root tips; RF = number of root forks.

To investigate the relationships among AGB, RB, RSR, RL, SA, AD, RV, RT, and RF of *P. ostii* under different container conditions, this study conducted a correlation analysis of the relevant indicators of *P. ostii*. As shown in Figure 3, The correlation coefficients between root-zone volume and aboveground biomass and root biomass were 0.909 and 0.895, respectively, and they were significant at the 0.01 level. This indicates that a larger root-zone volume contributes to an increase in the biomass of the aboveground parts and roots. It reflects that more space is beneficial for plants to acquire and allocate resources, thus promoting overall growth. The root-zone volume (RZV) was significantly positively correlated with RL, SA, RV, RT, and RF at different levels. This shows that under a larger root-zone volume, root structural characteristics such as length, surface area, volume, number of forks, and number of tips all increase, which is beneficial for enhancing the absorption capacity and support force of the roots. Aboveground biomass was significantly positively correlated with SA, RV, RT, and RF at the 0.05 or 0.01 level, indicating that a more developed root architecture contributes to the accumulation of aboveground biomass. This suggests that the growth and health of the aboveground parts depend to some extent on the expansion and architecture of the roots. The RSR was negatively correlated with indicators such as AGB, RL, SA, and RV. This shows that in the case of restricted root-zone volume, a higher RSR may reflect an adaptation strategy of plants to the restricted environment, reducing the growth of aboveground parts and concentrating resources to maintain the basic functions of the roots. Although the correlation between AD and RSR was not significant, there was a positive correlation in the trend. This positive correlation can be explained as follows: In small pots, due to space limitations, the roots of *P. ostii* increase the average diameter to enhance mechanical support and optimize root configuration in the limited space. At this time, the increase in underground biomass further magnifies the value of the root-to-shoot ratio. In addition, the experiment confirmed that the average root diameter showed the pattern of small pot > large pot > medium pot, with no significant difference between the large-pot and small-pot groups (*p* < 0.05). This indicates that although the direct contribution of average root diameter to the root-to-shoot ratio is limited, under extremely restricted root-zone conditions, increasing the root diameter may be an adaptation strategy of plants, which is consistent with the increasing trend of the root-to-shoot ratio.

#### 2.3.3. Principal Component Analysis (PCA) of Underground Root Architecture and Biomass

Principal component analysis (PCA) was performed on 10 data indices, with the first two principal components explaining a cumulative variance of 86.77%, which met the criterion of “cumulative variance contribution rate ≥ 85%” (Table 2). This indicates that these two principal components are the primary factors driving variations in *P. ostii* root architecture. Higher comprehensive scores of functional traits signify greater importance. Variables with high loadings on principal component 1, such as RV and AGB, reflect the growth potential of roots and shoots and resource acquisition capacity, highlighting their significance for overall plant growth. Variables on principal component 2, such as AD, are associated with structural adaptations of root systems, reflecting morphological adjustments and resource allocation strategies. RB, AGB, and RV—which showed significant correlations with RZV and had the highest comprehensive scores—play critical roles in evaluating overall plant growth and root architectural traits.

### 2.4. Fitting Curve Analysis of Root Architecture in P. ostii Under Root-Zone Restriction

**Figure 4:**
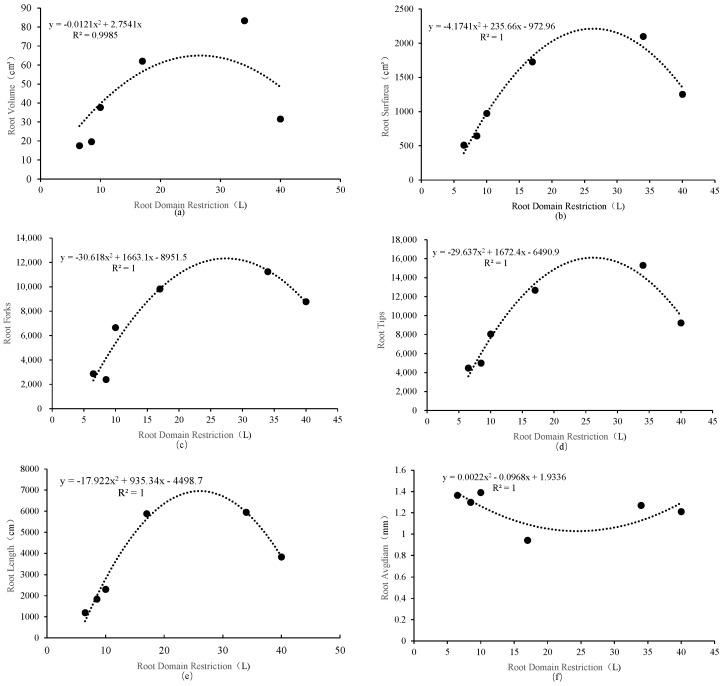
Fitting curve analysis of root-domain restriction on *P. ostii* root conformation. (**a**) root volume; (**b**) root surface area; (**c**) number of root forks; (**d**) number of root tips; (**e**) root length; (**f**) average root diameter.

Through the fitting analysis of the relevant data of the root system architecture of *P. ostii* under different root-zone restriction conditions within the set range (from 8.5 L to 34 L), different root system indexes showed significant patterns of change with the variation of root-zone restriction (Figure 4). The study found that the root length, the number of root tips, the root surface area, and the number of root bifurcations all first increased significantly with the increase in the container volume and then decreased slightly after reaching their peak values. Specifically, the root length reached its maximum value (7705.03 cm) when the container volume was 26.09 L. The peak values of the number of root tips, the root surface area, and the number of root bifurcations appeared at 28.21 L, 28.23 L, and 27.16 L, respectively. This indicates that when the volume of root-zone restriction is between 27 L–28 L, it has a certain promoting effect on the root tips and root surface area of *P. ostii*, suggesting that the optimal volume of root-zone restriction for *P. ostii* is 27 L to 28 L. In contrast, the root volume continued to increase with the increase in the container volume without showing a downward trend. While the average root diameter changed slightly within the research range, showing a relatively high degree of stability.

### 2.5. Effects of Root-Zone Restriction on Physiological and Biochemical Traits of P. ostii

#### 2.5.1. Impact of Root-Zone Restriction on the Antioxidant System

**Figure 5:**
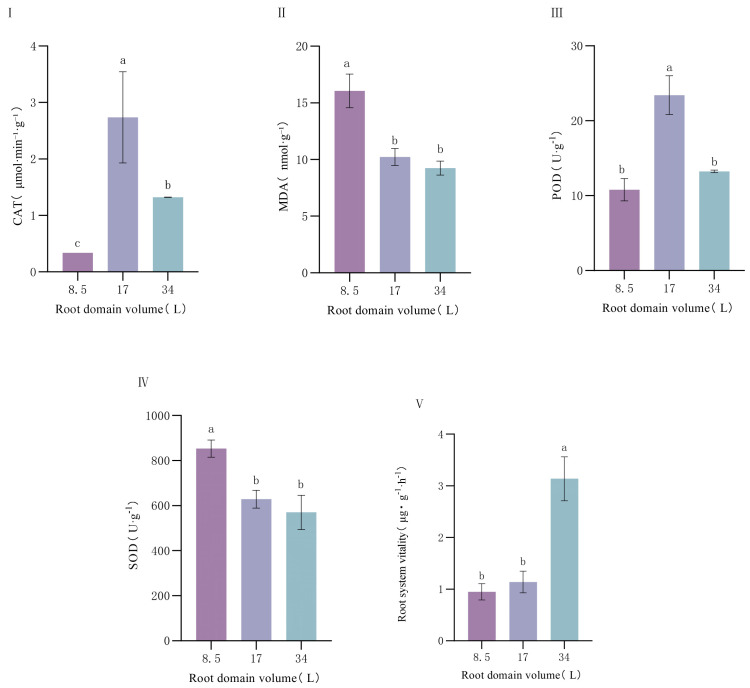
Effect of root-domain restriction on the antioxidant system of *P. ostii*. (**I**) catalase (CAT); (**II**) malondialdehyde (MDA); (**III**) peroxidase (POD); (**IV**) superoxide dismutase (SOD); (**V**) Root system vitality. Different lowercase letters indicate significant differences in plant antioxidant system in different root domain volume at 0.05 level.

Catalase (CAT), superoxide dismutase (SOD), peroxidase (POD) activities, malondialdehyde (MDA) content, and root viability exhibited significant differences under different root-zone conditions. CAT activity was lowest in the 8.5 L container, significantly lower than in the 17 L and 34 L containers, with catalase activity suppressed by 74.24% under root-zone restriction (Figure 5). In contrast, SOD activity showed an upward trend with decreasing container volume, peaking in the 8.5 L container and reaching the lowest level in the 34 L container—restricted root zones triggered a 49.74% significant upregulation of SOD activity. POD activity was highest in the 17 L container and lowest in the 8.5 L container, increasing by 53.85% in medium-volume containers compared to small ones. MDA content significantly increased in smaller containers, reaching the highest value in the 8.5 L treatment and the lowest in the 34 L treatment, with a 73.51% increase under root-zone restriction. Root viability gradually enhanced with increasing container volume, being strongest in the 34 L container, followed by 17 L, and lowest in the 8.5 L container.

Under different root-zone conditions, the expressions of antioxidant-related genes (*SOD*, *GPX8*, *GPX3*, and *CAT*) in *P. ostii* also showed different trends: The expression of the *SOD* gene was the highest in the container with a volume of 8.5 L and decreased rapidly as the container volume increased, which was consistent with the trend of *SOD* enzyme activity. The expressions of the *GPX8* and *GPX3* genes were the highest in the container with a volume of 17 L and relatively lower in the containers with volumes of 8.5 L and 34 L. The expression level of the *CAT* gene was the highest in the container with a volume of 34 L and the lowest in the container with a volume of 8.5 L. By integrating the results of the enzyme activities of the antioxidant enzyme system and the gene expressions, we found that *SOD* responded obviously to root-zone restriction. However, the expressions of *CAT* and *POD* at the gene level and their enzyme activities did not increase with the increase of root-zone restriction. This indicates that under the condition of root-zone restriction in *P. ostii*, the antioxidant enzyme system cannot completely eliminate oxygen free radicals, which easily leads to the accumulation of H_2_O_2_.

#### 2.5.2. Effects of Root-Zone Restriction on the Expression of Auxin-Related Genes in *P. ostii*

**Figure 6:**
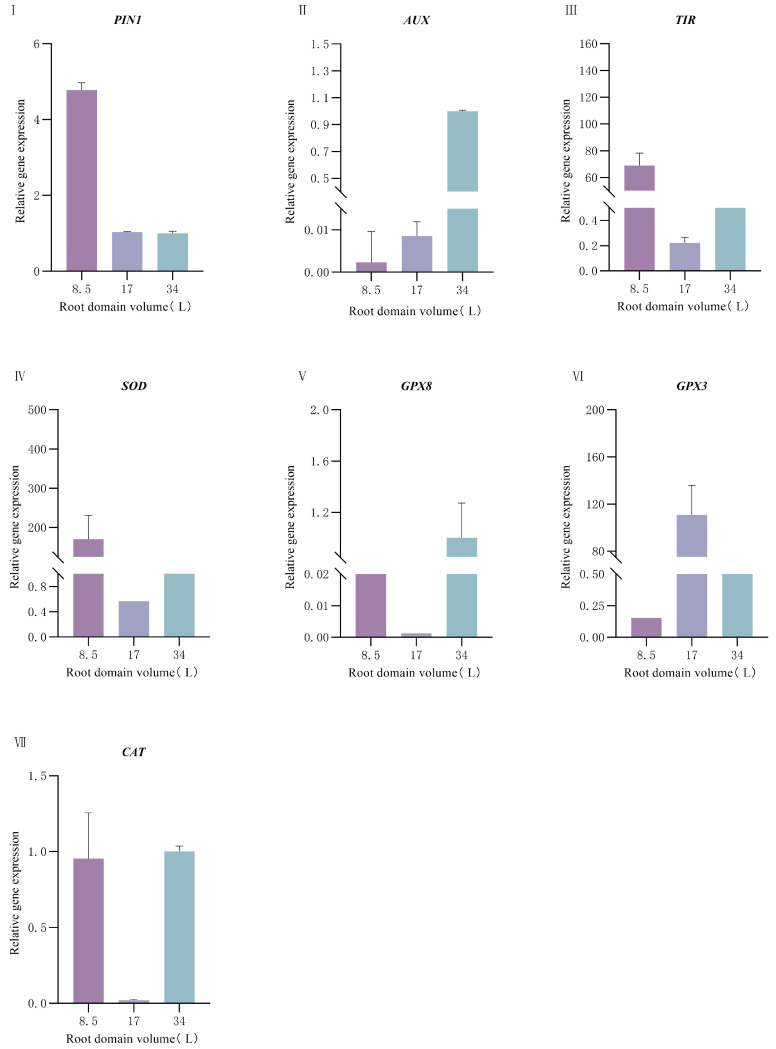
Effect of root-domain restriction on the expression of peony-related genes. (**I**) Relative gene expression of *PIN1*; (**II**) Relative gene expression of *AUX*; (**III**) Relative gene expression of *TIR*; (**IV**) Relative gene expression of *SOD*; (**V**) Relative gene expression of *GPX8*; (**VI**) Relative gene expression of *GPX3;* (**VII**) Relative gene expression of *CAT*.

Under different root-zone conditions, the expression levels of auxin-related genes (*PIN1*, *AUX*, and *TIR*) showed significant differences. The expression level of the *PIN1* gene decreased rapidly with the increase in container volume, reaching the lowest level in the 34 L container and the highest level in the 8.5 L container (Figure 6). The expression levels of the *AUX* and TIR genes were significantly higher in the 8.5 L container than in the 17 L and 34 L containers, showing a trend of decreasing as the container volume increased.

## 3. Discussion

### 3.1. Root Architectural Adaptation to Root-Zone Restriction

Root architecture critically governs soil resource acquisition and ecological functionality [23]. Under root-zone restriction (RZR), container specifications drive architectural reorganization, balancing soil resource efficiency with ecosystem stability [24,25,26]. Our data confirm that RZR redirects root growth orientation [27], while increased tip/fork density enhances absorption efficiency in confined spaces—consistent with Eissenstat and Yanai’s resource optimization principle [28]. Notably, *P. ostii* exhibited nonlinear architectural plasticity: root length, surface area, and fork number peaked at 26.09–28.23 L (Figure 4), maximizing absorption efficiency through elevated tip density. Beyond this threshold, plants prioritized structural stability via root diameter (AD) increase (Table 1), reflecting a trade-off between absorption area expansion and mechanical support. This adaptive strategy diverges fundamentally from woody-root species like grapes, which deploy high-density lateral roots under spatial constraints [21]. As a fleshy-rooted species, *P. ostii* relies on taproot thickening and cortical expansion [16], aligning with Poorter’s biomass-based container threshold (observed biomass: 1.7–2.3 g·L^−1^) [29]. Biomass allocation further revealed RZR-driven strategies: small containers (8.5 L) elevated root–shoot ratio (RSR) via “resource concentration” to roots; medium volumes (17 L) minimized RSR through “transitional compensation” toward reproductive growth; large containers (34 L) adopted an “expansion strategy” with 63% shoot biomass increase. This triphasic response contrasts with monotonic RSR shifts in fruit crops [8], underscoring *P. ostii*’s need to concurrently optimize flowering quality and storage organ development. These findings validate Eissenstat and Yanai’s root investment-return model [28], wherein restricted environments enhance per-unit-root efficiency. This research has shown that biomass allocation and root architecture are influenced by various abiotic factors, such as soil texture, nutrient availability, and moisture levels, which collectively affect root morphology and overall plant health [30]. Understanding these dynamics is essential for optimizing agricultural inputs and enhancing crop resilience in the face of environmental stressors. This study quantified the regulatory effects of root-zone restriction (RZR) on the root-to-shoot ratio (R/S) in *P. ostii*, yet the interaction between physical root confinement and nutrient availability remains coupled. Future work will employ a bivariate factorial design (container volume × nutrient gradient) to dissect their independent contributions to R/S: (1) using isotope tracing to resolve carbon allocation pathways; (2) integrating transcriptomics to identify interactive nodes between mechanical stress and nutrient signaling pathways; ultimately establishing a synergistic optimization framework for spatial and nutritional regulation.

### 3.2. Physiological and Molecular Response Mechanisms

Root-zone restriction (RZR) triggers integrated physiological and molecular adaptations in *P. ostii* beyond architectural modifications. Under RZR-induced oxidative stress, root activity declined while malondialdehyde (MDA) content surged by 55% (8.5 L vs. 34 L), confirming ROS imbalance and membrane damage [30,31]. Antioxidant enzymes exhibited compartmentalized regulation: SOD activity increased by 42% in restricted roots (8.5 L), scavenging O_2_^−^ radicals [32,33,34], while CAT activity decreased by 31%, impairing H_2_O_2_ detoxification [31,35,36]. POD peaked at 17 L, indicating redox buffering at moderate stress [37,38,39]. This disrupted SOD–CAT–POD cascade [40] validated H_2_O_2_ accumulation as a key metabolic constraint. Critically, RZR-induced mechanical confinement activated auxin signaling as a compensatory response. The upregulation of *PIN1* and *AUX1* genes (Figure 6) promoted lateral root initiation and enhanced root tip density (Figure 2), aligning with the mechanical stress-auxin feedback loop observed in root development [23,41]. Although cytokinin/ethylene pathways were not assessed, their roles in RZR adaptation warrant future investigation. Importantly, non-RZR variables (light, irrigation, homogeneous volcanic soil: native soil substrate (1:1 *v*/*v*)) were strictly controlled (Section 4.2 and Section 4.3), and PCA confirmed root architectural variations (86.77% cumulative variance) were primarily driven by container volume (Table 2), supporting RZR as the dominant factor. Concurrently, RZR activated gene networks governing stress tolerance and development. Antioxidant genes (*SOD*, *GPX3/8*, and *CAT*) were upregulated in 8.5 L roots, enhancing ROS quenching (*SOD*: O_2_^−^ → H_2_O_2_ [33]; *GPX*: peroxide reduction [42]; *CAT*: H_2_O_2_ decomposition [43]). The upregulation of these genes enhances the antioxidant capacity of *P. ostii* under root-zone restriction, improving plant survival and adaptability. Additionally, genes closely related to root growth and development (*PIN1* and *AUX1*) were significantly upregulated in small containers, suggesting that plants optimize resource absorption by accelerating lateral root development in limited space—a regulatory mechanism closely associated with the key role of auxin signaling in root development [44,45,46]. These molecular shifts synergistically improved nutrient acquisition under spatial constraints [41,47].

### 3.3. Horticultural Implications and Optimization Strategies

Root-zone restriction (RZR) technology demonstrates significant cross-species efficacy in enhancing fruit quality through improved nutrient partitioning, as evidenced in grape anthocyanin accumulation [8], tomato metabolic regulation [13], and cotton photosynthate allocation [48]. For *P. ostii*, our study reveals that RZR fundamentally restructures root architecture plasticity, with optimal efficiency occurring at 26.09–28.23 L where root length, tip density, and bifurcation peak (Figure 4). This volume threshold provides a quantitative basis for balancing ornamental yield (e.g., 63% AGB increase in 34 L) and root functionality, contrasting with Liu et al.’s findings on nitrogen-hormone interactions under RZR [49]. We propose a dynamic restriction strategy integrating three innovations. Phased container management: 8.5 L (seedling) → 27 L (juvenile) → 34 L (pre-flowering) to sequentially induce high RSR (44.8% increase), optimize root branching, and enhance floral quality. Substrate engineering: High-density substrates (bulk density > 1.2 g/cm^3^) for primary root thickening at the seedling stage, transitioning to aerated media (porosity > 35%) for fine root proliferation at maturity. Perlite-vermiculite mix (3:1): Provides essential Ca^2+^/Mg^2+^ (CEC = 28 cmol/kg) while meeting the aeration demands of fleshy roots. Validation trials confirm this approach increases fresh root biomass by 31% and extends flowering by 5–7 days compared to static cultivation. Crucially, exceeding the 28 L threshold diminishes marginal returns, while RSR > 2.57 or MDA > 4.5 nmol/g indicates oxidative stress requiring container upscaling. These protocols resolve the “dilution effect” in oversized containers while preventing resource limitation in restricted volumes, advancing precision horticulture for fleshy-rooted species. In landscape applications, planting spaces can be strategically designed based on peony root-zone requirements of *P. ostii* identified in this study. By integrating substrate modification and root management, plants can achieve optimal root expansion, ensuring stable nutrient and water absorption during growth, prolonging the growth cycle, and reducing the repotting frequency. Beyond targeted root-zone design, future practices could incorporate precision fertilization and soil management to enhance root development and resource use efficiency. This approach would improve the overall productivity of crops like *P. ostii* under stress conditions while reducing dependency on chemical fertilizer inputs [50].

## 4. Materials and Methods

### 4.1. Experimental Site Description

The Shanghai Chenshan Botanical Garden (121.182501° E, 31.075935° N), located in Songjiang District, Shanghai, is situated in the subtropical monsoon climate zone, characterized by distinct seasons. The annual average temperature is approximately 15.6 °C, the frost-free period lasts about 230 days, annual sunshine duration is 1817 h, and the annual average precipitation is 1200 mm. The experimental site was located in the field nursery of the East Horticulture Station at Chenshan Botanical Garden.

### 4.2. Experimental Materials

Three-year-old *P. ostii* with uniform height and growth vigor were selected as the research material. Plants were cultivated in a medium consisting of volcanic stone mixed with native soil at a volume ratio of 1:1 (volcanic stone:native soil) and planted in containers of six sizes: 6.5 L, 8.5 L, 10 L, 17 L, 34 L, and 40 L. The volumes of 8.5 L, 17 L, and 34 L represent the most commonly used container sizes during transplanting of divided plants in the initial cultivation phase of tree peonies. During the cultivation period, plants in each treatment group were grown under identical light and humidity conditions, with regular watering and fertilization to ensure normal growth.

### 4.3. Experimental Design

Three container sizes: 8.5 L (small pot), 17 L (medium pot), and 34 L (large pot) were utilized for the root-zone restriction experiment, with 15 pots per treatment to ensure sufficient sample size and reliability of results. Plants were subjected to a completely randomized block design featuring three blocks of 15 pots each, with sampling planned for 2024, 2025, and 2026. *P. ostii* were planted in October 2022, and at the end of the full-bloom stage in May 2024, three pots were randomly selected from each treatment. All experimental plants were harvested, separated into aboveground and underground parts, and fresh weights were recorded. *P. ostii* samples were then subjected to blanching at 105 °C followed by drying at 75 °C to constant weight. Root systems were scanned using an Epson Expression 120000 XL scanner (Epso, Suwa, Japan), and root architecture was analyzed with WinRHIZO Pro2019 software (Regent Instruments Inc., Quebec City, QC, Canada).

Physiological and biochemical assays: Root samples (0–5 cm from the root tip) were collected to avoid mechanical damage, rapidly frozen in liquid nitrogen, and stored at −80 °C to prevent enzyme degradation. Root viability was measured using the 2,3,5-triphenyltetrazolium chloride (TTC) method. For enzyme activity assays, roots were cleaned, ground in liquid nitrogen, and assayed for peroxidase (POD), superoxide dismutase (SOD), catalase (CAT), and malondialdehyde (MDA) content following the protocols provided by Suzhou Keming Biotechnology Co., Ltd., Suzhou, China.

Gene expression analysis: Total RNA was extracted from root tissues using TRIzol reagent (Thermo Fisher Scientific, Beijing, China). Using oligo (dT) as a primer, the first-strand cDNA was synthesized with the RETROscript kit (Invitrogen, Beijing, China). Three genes related to auxin transport and signal transduction, namely auxin efflux carrier component 1 (*PIN1*), auxin transporter-like protein (*AUX1*), and auxin receptor protein (*TIR1*), were detected by reverse transcription polymerase chain reaction (RT-PCR) and quantitative polymerase chain reaction (Q-PCR). Four genes are related to the elimination of oxygen free radicals, namely superoxide dismutase (SOD), catalase (CAT), and peroxidases *GPX3* and G*PX8*. The relevant specific primers are shown in Table 3. Each Q-PCR reaction system (25 μL) contained 25 ng of template cDNA, 12.5 μL of 2 × SYBR Green I fluorescent dye premix buffer (Transgene, Shanghai, China), and 300 nM of forward and reverse primers. The reaction was carried out on an ABI PRISM 5700 sequence detector (Applied Biosystems, Waltham, MA, USA), and the cycling conditions were as follows: pre-denaturation at 94 °C for 4 min, followed by 40 cycles, with each cycle consisting of denaturation at 94 °C for 30 s, annealing at 58 °C for 30 s, and extension at 72 °C for 30 s. *ACTIN1* (XM_024302219.2) was selected as the internal reference gene (Table 3). Each gene was detected three times. The amplification efficiency of all PCR reactions was above 95%. The median coefficient of variation of the repeated samples (based on the calculated quantities) was 6%.

### 4.4. Data Analysis

Preliminary data processing was carried out using Microsoft Excel 2021. One-way analysis of variance (ANOVA) and plotting were performed using GraphPad Prism 10.1.2. Principal component analysis (PCA) of root parameters was conducted using SPSS 27. Correlation analysis between the aboveground and underground parts of plants under different treatments was carried out using Origin 2022.

The root-to-shoot ratio (RSR), a key plant physiological indicator, is defined as the ratio of the dry weight of the plant roots to the dry weight of the aboveground parts. The calculation formula is:

$$\frac{R}{S}=\frac{Dry\;weight\;of\;roots\;(g)}{Dry\;weight\;of\;aboveground\;parts\;(g)}$$

## 5. Conclusions

This study demonstrates that root-zone restriction (RZR) fundamentally reshapes *P. ostii*’s adaptive strategies across morphological, physiological, and molecular levels. While these findings provide mechanistic insights under Shanghai’s subtropical monsoon climate, restricted root space (e.g., 8.5 L) triggered a 44.8% increase in root-to-shoot ratio and elevated SOD activity by 49.74%, indicating carbon reallocation to roots and ROS–auxin crosstalk as key survival mechanisms. Crucially, optimal root efficiency occurred at 26–28 L, where root length, tip density, and bifurcation peaked—providing a quantitative threshold for balancing ornamental value and root function. We propose a stepwise container strategy (8.5 L → 27 L → 34 L) to enhance root architecture plasticity while reducing cultivation costs. These findings establish RZR as a vital tool for horticultural optimization in woody plants, with ROS–auxin dynamics serving as biomarkers for stress resilience. This study revealed the significant influence of root-zone restriction on morphological, physiological, and molecular traits. Future research should validate these responses across diverse climatic zones and soil types to assess broader applicability. Specifically, investigations should prioritize interactions between root development and environmental variables to cultivate stress-resilient plant varieties. Additionally, longitudinal studies assessing multidimensional environmental impacts on root systems could also provide critical data for predicting future multiphysics-coupled efficiency and sustainability in energy production, plant yield, carbon sequestration, and temperature mitigation.

## Figures and Tables

**Table 1:** Characterization of belowground root system configuration and biomass variability.

Quantitative Character	Minimum Value	Maximum Value	Mean Value	Standard Deviation	Coefficient of Variation/%
AGB (g)	6.48	76.37	33.87	25.30	74.68%
RB (g)	16.68	97.75	47.15	32.86	69.70%
RSR	0.68	2.57	1.60	0.53	32.93%
RL (cm)	922.76	8564.30	4554.12	2569.72	56.43%
SA (cm^2^)	368.76	2885.23	1491.71	797.69	53.47%
AD (mm)	0.88	1.57	1.17	0.22	18.48%
RV (cm^3^)	11.73	130.25	48.36	35.31	73.01%
RT	2486.00	22,344.00	10,992.89	6409.14	58.30%
RF	1816.00	14,271.00	7817.22	4592.95	58.75%

AGB = aboveground biomass; RB = root biomass; RSR = root–shoot ratio; RL = root length; SA = root surface area; AD = average root diameter; RV = root volume; RT = number of root tips; RF = number of root forks.

**Table 2:** Principal component analysis of conformational characteristics and biomass of underground root systems.

Functional Trait	Principal Component 1	Principal Component 2	Comprehensive Score	Comprehensive Ranking	Communality
RB	0.878	0.374	0.324	1	0.911
RZV	0.920	0.276	0.324	2	0.923
RV	0.936	0.225	0.321	3	0.927
AGB	0.888	0.297	0.316	4	0.876
SA	0.981	−0.127	0.286	5	0.978
RF	0.960	−0.199	0.270	6	0.961
RT	0.931	−0.181	0.264	7	0.900
RL	0.928	−0.312	0.244	8	0.958
AD	−0.194	0.933	0.069	9	0.907
RSR	−0.548	0.187	−0.144	10	0.335
Eigenvalue (math.)	2.689	1.202			
contribution rate	72.310	14.460			
Cumulative contribution rate	72.310	86.770			

**Table 3:** Table of primers for target genes and GenBank sequence identifiers.

Gene Symbol	Sequence of Primer	GenBank Sequence ID
*ACTIN1*	F: 5′-TGGATTTGCTGGAGATGATGC-3′ R: 5′-TCCATATCATCCCAGTTGCTC-3′	XM_024302219.2
*PIN1*	F: 5′-GACTTCTACCACGTCATGAC-3′ R: 5′-GAGGAAGCGGGTGTTCATG-3′	KF543362.1
*AUX1*	F: 5′-CTGTGCTTCCAATCAAGTGG-3′ R: 5′-ATCAAGCACTTCAAACCACTG-3′	XM_024333122.2
*TIR1*	F: 5′-ACGTGTTCTCGTTCCTGCA-3′ R: 5′-ATTGAAGTCGGCGAAGTGAG-3′	XM_024306982.2
*SOD*	F: 5′-AGAAGCACCACCAGGCTTA-3′ R: 5′-CAGTGGTTTCAACCACAACC-3′	XM_024322895.2
*GPX8*	F: 5′-ACTGATTGTCAATGTTGCTTCC-3′ R: 5′-ATGGAGCAGCATTATCACCAT-3′	XM_050523083.1
*GPX3*	F: 5′-TACGAATTCACTGTCAAGGATATTC-3′ R: 5′-GTTTCCTGGCTCTTGTCCTG-3′	XM_062138578.1
*CAT*	F: 5′-ATGGATCCTTACAAGTACCGC-3′ R: 5′-AATCCCTTTGCACTGGCTC-3′	XM_062170099.1

## Data Availability

The original contributions presented in this study are included in the article. Further inquiries can be directed to the corresponding author.
